# Kidney Dysfunction, Biochemical Changes, DNA Alteration, and MAPKs Regulation Following Chronic Exposure to Regular and Occasional Hookah Smoke in Mice

**DOI:** 10.1155/omcl/6069542

**Published:** 2026-01-07

**Authors:** Naserddine Hamadi, Anas Nemmar, Sumaya Beegam, Nur Elena Zaaba, Ozaz Elzaki, Abderrahim Nemmar

**Affiliations:** ^1^ Department of Environmental Sciences and Sustainability, College of Natural and Health Sciences, Zayed University, Abu Dhabi, P.O. Box 144534, UAE, zu.ac.ae; ^2^ College of Medicine, Gulf Medical University, Ajman, UAE, gmu.ac.ae; ^3^ Department of Physiology, College of Medicine and Health Sciences, United Arab Emirates University, Al Ain, P.O. Box 15551, UAE, uaeu.ac.ae; ^4^ Zayed Center for Health Sciences, United Arab Emirates University, Al Ain, UAE, uaeu.ac.ae

**Keywords:** DNA damage, hookah smoke, inflammation, kidney, oxidative stress

## Abstract

Regular hookah smoking (Reg‐HS) has become a major global public health issue, linked to significant health risks, including kidney damage. A less frequent pattern of use, known as occasional hookah smoking (Occ‐HS), is also common; however, there has been little progress in understanding the direct impact of Occ‐HS on kidneys. To investigate how varying frequencies of HS inhalation affect the kidney, we exposed mice to nose‐only HS under two regimens, occasional (30 min once weekly) and regular (30 min five times per week) for a duration of 6 months. This study explored the impact on renal damage, inflammatory responses, oxidative stress levels, genotoxicity, and mitochondrial activity as well as the possible modulation of MAPK signaling pathway. Both Occ‐HS and Reg‐HS led to a marked elevations in plasma levels of urea and creatinine (*p* < 0.05–*p* < 0.0001). Additionally, concentrations of kidney injury molecule‐1 (KIM‐1) and neutrophil gelatinase‐associated lipocalin (NGAL) were significantly increased in both groups (*p* < 0.01–*p* < 0.0001). Notably, only the Reg‐HS regimen induced a substantial rise in plasma levels of indoxyl sulfate, cystatin C, and adiponectin (*p* < 0.01–*p* < 0.0001). Similarly, relative to the control group, mice subjected to Reg‐HS exposure exhibited significantly elevated levels of proinflammatory cytokines, tumor necrosis factor‐*α*, and interleukin‐6 (*p* < 0.0001). Exposure to either Occ‐HS or Reg‐HS caused significant increase in interleukin‐1*β* (*p* < 0.05, *p* < 0.0001), thiobarbituric acid reactive substances (TBARS; *p* < 0.05, *p* < 0.0001) compared with air‐exposed mice. Our findings revealed that Occ‐HS inhalation triggered only a decrease in superoxide dismutase (SOD) activity (*p* < 0.001). On the other hand, nitric oxide (NO; *p* < 0.001), SOD (*p* < 0.0001), and Glutathione (GSH; *p* < 0.0001) levels were significantly decreased in Reg‐HS group. Furthermore, DNA damage marker, 8‐Hydroxy‐2^′^‐deoxyguanosine was significantly augmented in both regimens (*p* < 0.0001). Exposure to both regimens resulted in significant elevation in mitochondrial complexes I, II and III, and IV (*p* < 0.0001). Increased expression of activation of mitogen‐activated protein kinases (MAPKs) was observed exclusively in the Reg‐HS group, as evidenced by increased levels of p‐JNK, p‐p38, and p‐ERK (*p* < 0.001–*p* < 0.0001). In conclusion, our study is the first to demonstrate that despite the significant differences in the amount of smoke inhaled, both Occ‐HS or Reg‐HS inhalation deteriorate kidney function and induce oxidative damage, inflammatory response, DNA injury, and mitochondrial impairment with modulation of the MAPK signaling. These findings highlight the importance of further research into the public health risks associated with occasional hookah smoking.

**Local Terms Used in the Manuscript**

This manuscript uses the term Hookah smoking to describe the inhalation of tobacco through a waterpipe device. The standard term for this practice is waterpipe tobacco smoking. This method involves passing smoke through water before it is inhaled. Also, it is known by various names across different regions, including hookah, shisha, narghile, hubble‐bubble, or argileh.

## 1. Introduction

Hookah smoking (HS) has traditionally utilized for generations as a means of enjoying tobacco through inhalation in Eastern Mediterranean and across various regions of Asia and Africa [1]. Given the dramatic increase in the consumption of HS worldwide [2]. Many studies have undisputedly established that HS presents equal or potentially greater health risks as compared to cigarette smoke (CS), owing to its prolonged smoking sessions and the presence of similar harmful toxicants [3, 4].

It is well established that there is a strong correlation between smoking intensity and the associated risks of developing fatal conditions, like chronic obstructive lung conditions, cardiovascular ischemia, and pulmonary carcinoma [5–7]. Worth noting that, a growing portion of smokers are non‐daily users, called as non‐daily smokers, representing upto 38% of US adult smokers [8]. Nevertheless, available evidence remains scarce that explore the harmful impact of occasional tobacco use on health. Unexpectedly, in a survey conducted in France, 34% of respondents believed that consuming upto 10 cigarets daily carries no risk of developing lung cancer [9].

It has been indicated by a systematic review and meta‐analysis that lesser cigarette consumption may lower the risk of lung cancer, but the overall risk remains significantly high [10]. Additionally, studies have shown that smoking even a single cigarette daily markedly elevates the risk of coronary heart disease and stroke, and it approximately doubles if one smokes 20 cigarettes a day [11]. A recent study among US adults, revealed that lifelong light smoking is linked to a higher case fatality rate for cardiovascular diseases [12]. Moreover, occasional smoking among Finnish men has been associated with a 50% increase in cardiovascular disease mortality [13].

The kidneys are fundamentally structured to filter plasma, reabsorb necessary substances, and eliminate waste products. These essential functions are crucial for regulating body fluid osmolality and removing metabolic waste [14]. The kidney is vulnerable to the harmful effects of various toxins, including those found in tobacco products. The injurious effects of daily CS on the kidneys have been demonstrated on a wide scale, including in vitro, in vivo, and human studies [15–19].

Our recent research has shown that the kidneys are equally susceptible to the damaging impacts of regular HS (Reg‐HS) [20, 21]. However, there is still a lack of data comparing the toxicity of occasional hookah smoke (Occ‐HS) versus Reg‐HS exposure. We have recently reported the deleterious effects of Occ‐HS on the cardiovascular system and lung [22–24].

Accordingly, the present study was designed to assess the effects of 6‐month nose‐only exposure to either Occ‐HS (30 min/day, 1 day/week) or Reg‐HS (30 min/day, 5 days/week) on kidney injury, oxidative damage, inflammation, DNA integrity, and mitochondrial activity. Furthermore, we investigated whether downstream signaling pathways, particularly mitogen‐activated protein kinases (MAPKs), are activated during this process.

## 2. Materials and Methods

### 2.1. Experimental Protocols and Treatments

Ethical approval for this study was granted by the UAEU Animal Ethics Review Committee (Approval No. ERA_2017_5625). All experimental procedures were carried out under the guidance of the Committee’s approved protocols.

A cohort of male BALB/c mice, sourced from the UAEU, College of Medicine and Health Sciences, Animal Facility, were maintained under standardized environmental conditions: 12 h of light beginning at 06:00, followed by 12 h of darkness. Throughout the study, animals had unrestricted access to standard chow and water. After 7 days of acclimatization, mice were randomized into three experimental groups, air, Occ‐HS and Reg‐HS. Each session lasted 30 min/day. Control mice were exposed to air only. Regarding Occ‐HS, mice were exposed to HS, 1 day/week for 6 months [22, 23] and for Reg‐HS group, 5 days/week for 6 months. HS was introduced at 60 s intervals in a controlled manner, consisting of 2 s puff of HS, followed by a 58 s of fresh air period of filtered air to ensure controlled, repeatable exposure.

To replicate traditional HS, apple‐flavored tobacco (Al‐Fakher, Ajman, UAE) was ignited using charcoal disc. The resulting smoke was filtered through water before being directed into the exposure chamber. Mice were softly restrained and connected to the exposure apparatus following an established protocol [25, 26]. A computer‐regulated delivery system (InExpose, Scireq, Canada) facilitated controlled nose‐only exposure of mice to HS, ensuring consistent and reproducible inhalation dynamic.

### 2.2. Blood Collection and Biochemical Analysis

On the day of sample collection, mice were first anesthetized via intraperitoneal injection of sodium pentobarbital (60 mg/kg). Blood was drawn from the inferior vena cava into heparinized tubes, followed by centrifugation at 900 × *g* for 15 min at 4°C to isolate plasma. The resulting plasma was aliquoted and stored at −80°C for subsequent biochemical analyses. Final euthanasia was performed using a sodium pentobarbital overdose. Kidneys were rapidly excised, snap‐frozen in liquid nitrogen, and preserved at −80°C until further processing. Tissue homogenization and preparation for biochemical assays were conducted according to established protocols [27, 28].

### 2.3. Measurement of Urea and Creatinine Concentrations in the Plasma

The plasma levels of creatinine and urea were assessed spectrophotometrically according to the manufacturer (Roche Diagnostics, Indianapolis, IN, USA).

### 2.4. Assessment of Kidney Injury Markers (Cystatin C, Neutrophil Gelatinase‐Associated Lipocalin [NGAL], Adiponectin, Indoxyl Sulfate, and Kidney Injury Molecule‐1 [KIM‐1]), Proinflammatory Cytokines (Interleukin‐1*β* [IL‐1*β*], Tumor Necrosis Factor‐*α* [TNF‐*α*], and Interleukin‐6 [IL‐6]), DNA Damage Marker, and 8‐Hydroxy‐2‐Deoxyguanosine (8‐OHdG) in the Kidney Tissue Homogenate

The kidney concentration of indoxyl sulfate was assessed by enzyme‐linked immunosorbent assay (ELISA) (My BioSource, CA, USA). Furthermore, the concentrations of KIM‐1, cystatin C, NGAL, adiponectin, TNF‐*α*, IL‐6, IL‐1*β*, and 8‐OHdG were measured using ELISA kits (R and D systems, Minneapolis, MN, USA).

### 2.5. Measurement of Glutathione (GSH), Nitric Oxide (NO), Thiobarbituric Acid Reactive Substances (TBARS), and Superoxide Dismutase (SOD) Levels in Kidney Tissue Homogenate

Lipid peroxidation levels were quantified using TBARS assay, calibrated against a malondialdehyde standard as well as GSH concentrations (Sigma–Aldrich, St. Louis, MO, USA). SOD enzymatic activity were determined following the manufacturers’ standardized protocols (Cayman Chemical, Ann Arbor, MI, USA).

### 2.6. Measurement of Mitochondrial Complexes I, II and III, and IV (C) Activities in Kidney Tissue Homogenate

Mitochondrial fractions were purified from mice renal tissue via differential centrifugation technique [23]. Kidneys were homogenized in mitochondrial isolation buffer (sucrose [0.32 M], EDTA [1 mM], tris base [10 mM]) using a Dounce homogenizer. Following centrifugation at 1000 rpm for 10 min at 4°C, the supernatant was collected and spun at 15,000 rpm for 15 min at 4°C. Mitochondrial pellets were resuspended in buffer and stored at −80°C for subsequent analyses.

Complex I enzymatic activity was measured following previously established protocols [29, 30]. In brief, 25 *μ* g of mitochondrial protein were added to each well of a 96‐well plate containing distilled water, BSA (50 mg/mL), KCN (10 mM), PBS (0.5 M, pH 7.5), and NADH (10 mM). To evaluate the assay specificity, a parallel set of wells was prepared with the same reagents plus rotenone (1 mM), a known complex I inhibitor. Following the addition of ubiquinone (5 mM), NADH oxidation was monitored spectrophotometrically at 340 nm for 10 min. The estimation of the complex I enzymatic activity was done as mentioned previously [29, 30].

To measure the activities of mitochondrial complexes II and III, we followed a previously established protocol [29, 30]. In short, we added to each well of a 96‐well plate a mixture containing distilled water, BSA (50 mg/mL), KCN (10 mM), PBS (0.5 M, pH 7.5), NADH (10 mM), and 25 *μ* g of isolated mitochondrial proteins. After a 10 min warm‐up at 37°C on orbital shaker, the start of the reaction was induced by the supplementation of oxidized cytochrome C (1 mM) and the augmentation in the absorbance measured over a 5 min span at 550 nm. The specificity of the assay was evaluated using control wells containing the same reagents and the samples were added along with antimycin A (1 mg/mL) or malonate (1 M). The estimation of the complexes II and III activities was done as mentioned previously [29, 30].

The complex IV activity was quantified following the methodology described in prior reports [29, 30]. Briefly, a 96‐well plate was prepared with distilled water, reduced cytochrome C (1 mM), and PBS (0.1 M). Baseline absorbance at 550 nm was recorded for 2 min. The reaction was initiated by adding 25 *μ* g of isolated mitochondria, and the subsequent decrease in absorbance at 550 nm was monitored for 10 min. To verify assay specificity, a parallel reaction containing identical reagents and samples was supplemented with KCN (10 mM). Complex IV activity was calculated using the same formula employed for complexes II and III [29, 30].

### 2.7. Measurement of Expression p‐p38, p‐JNK, and p‐ERK

To quantify the expression of p‐JNK, p‐ERK, and p‐p38 we used western blot technique, as previously described [21]. At 4°C, the kidney homogenates were centrifuged for 20 min. To measure total protein in the kidney homogenate, BCA kit was used (Thermo Scientific, Waltham, MA, USA).

Using 10% SDS‐PAGE, 35 µg of protein were separated and transferred to PVDF membranes. Membranes were blocked with 5% non‐fat milk for 1 h at room temperature. Rabbit monoclonal antibodies against p‐ERK (1:2000), p‐JNK (1:2000), and p‐p38 (1:2000) (Santa Cruz, Dallas, TX, USA) were applied and incubated overnight at 4°C. After three 10 min washes, membranes were incubated with goat anti‐rabbit IgG HRP‐conjugated secondary antibody (Abcam, Hong Kong, China) for 2 h at room temperature.

Blots were developed using the Pierce ECL Plus kit (Thermo Scientific) and imaged with the Typhoon FLA 9500 system (GE Healthcare) for densitometric analysis. *β*‐actin (1:10,000, Santa Cruz, Dallas, TX, USA) was used as a loading control.

### 2.8. Statistical Analysis

Data are presented as mean ± SEM. Statistical analysis was conducted using one‐way ANOVA followed by Holm–Sidak’s post hoc test in GraphPad Prism v7 (GraphPad Software, San Diego, CA, USA). Statistical significance was set at *p*  ≤ 0.05.

## 3. Results

### 3.1. Concentrations of Urea, Creatinine, and Indoxyl Sulfate in the Plasma

A significant increase in the plasma levels of creatinine and urea were seen after Occ‐HS exposure (*p* < 0.05–*p* < 0.0001). Similarly, exposure to Reg‐HS induced an augmentation in the levels of urea as well as creatinine (*p* < 0.0001). However, only Reg‐HS caused a higher level of indoxyl sulfate (*p* < 0.0001). Furthermore, we observed a significant difference between the exposure to either Occ‐HS or Reg‐HS in the concentrations of urea, creatinine, and indoxyl sulfate (*p* < 0.01–*p* < 0.0001) (Figure 1).

Figure 1Plasma concentrations of urea (A), creatinine (B), and indoxyl sulfate (C) after 6 months of exposure to either air (control), occasional hookah smoke (Occ‐HS), or regular HS (Reg‐HS). Data are presented as mean ± SEM (*n* = 8).

**Figure:**
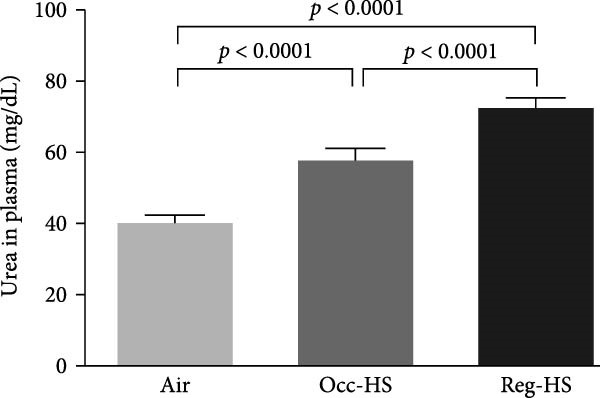
(A)

**Figure:**
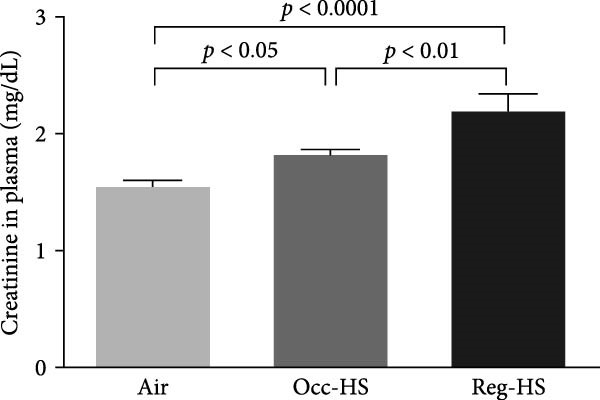
(B)

**Figure:**
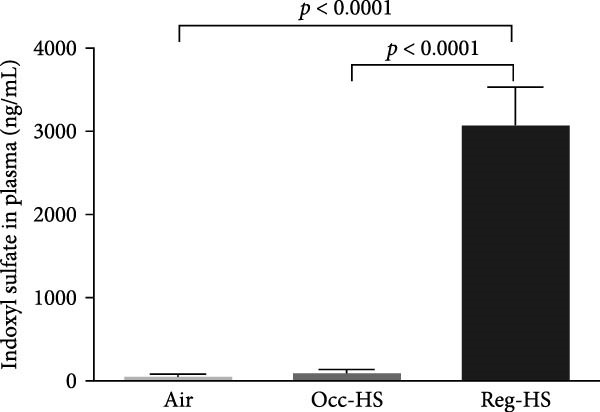
(C)

### 3.2. Levels of Kidney Injury Markers NGAL, Cystatin C, KIM‐1, and Adiponectin in the Kidney Tissue Homogenate

Figure 2 illustrates how the exposure to both regimens Occ‐HS or Reg‐HS impacted the levels of KIM‐1, NGAL, cystatin C, and adiponectin (Figure 2A–D). Exposure to Occ‐HS led to a considerable rise in KIM‐1 and NGAL concentrations in the kidney compared to air group (*p* < 0.01–*p* < 0.001). The chronic exposure to Reg‐HS caused a significant elevation in KIM‐1, NGAL, cystatin C, and adiponectin (*p* < 0.01–*p* < 0.0001). Moreover, the levels of all four measured parameters differed significantly between the Occ‐HS and Reg‐HS exposure groups (*p* < 0.01–*p* < 0.0001).

Figure 2Kidney injury molecule‐1 (KIM‐1, A), neutrophil gelatinase‐associated lipocalin (NGAL, B), cystatin C (C), and adiponectin (D) concentrations in kidney tissue homogenates after 6 months of exposure to either air (control), occasional hookah smoke (Occ‐HS), or regular HS (Reg‐HS). Data are presented as mean ± SEM (*n* = 8).

**Figure:**
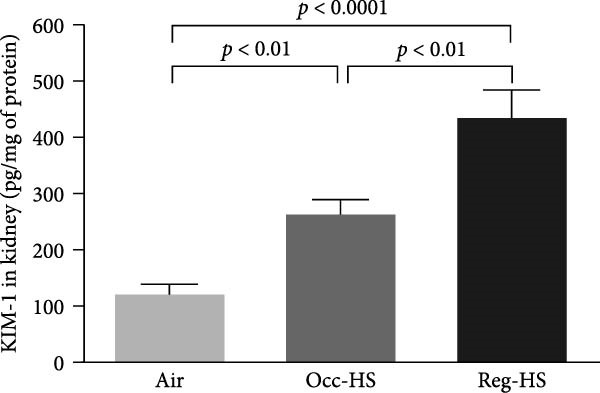
(A)

**Figure:**
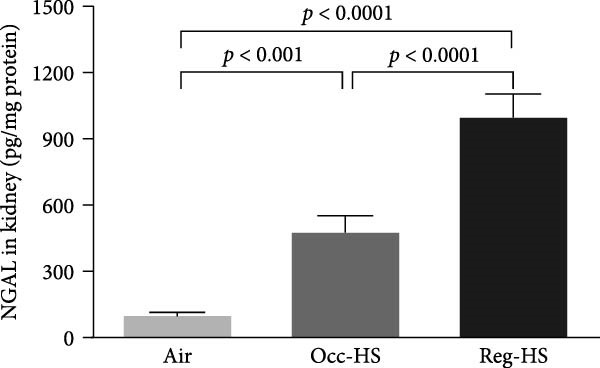
(B)

**Figure:**
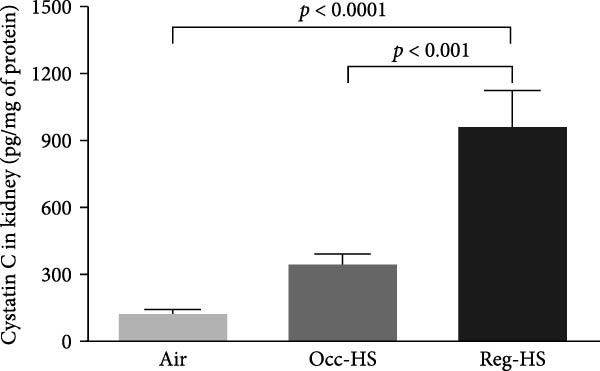
(C)

**Figure:**
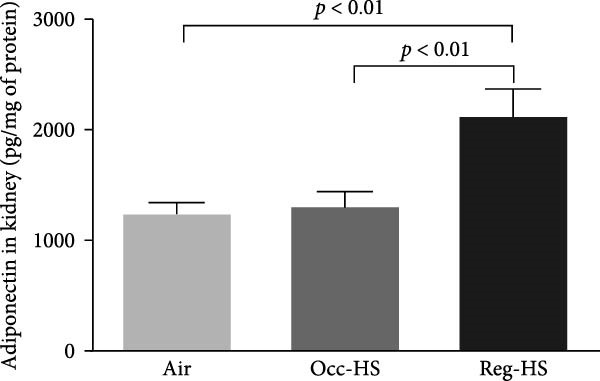
(D)

### 3.3. Concentrations of Proinflammatory Cytokines in the Kidney Tissue Homogenate

The levels of TNF‐*α*, IL‐6, and IL‐1*β* measured by ELISA were presented in Figure 3. Our results show 6 months Occ‐HS exposure increased only the concentration of IL‐1*β* in the kidney homogenate (*p* < 0.05). In contrast, TNF‐*α*, IL‐6, and IL‐1*β* levels were augmented significantly after Reg‐HS inhalation compared to air group (*p* < 0.0001). Moreover, TNF‐*α*, IL‐6, and IL‐1*β* concentrations were significantly different in Occ‐HS vs. Reg‐HS groups (*p* < 0.0001).

Figure 3Concentrations of tumor necrosis factor‐*α* (TNF‐*α*; A), interleukin‐6 (IL‐6; B), and interleukin‐1*β* (IL‐1*β*; C) in kidney tissue homogenates after 6 months of exposure to either air (control), occasional hookah smoke (Occ‐HS), or regular HS (Reg‐HS). Data are presented as mean ± SEM (*n* = 8).

**Figure:**
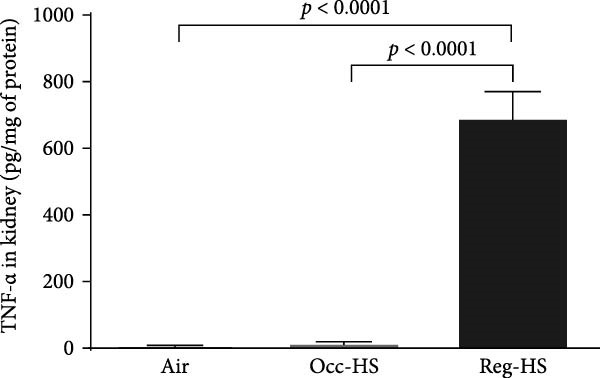
(A)

**Figure:**
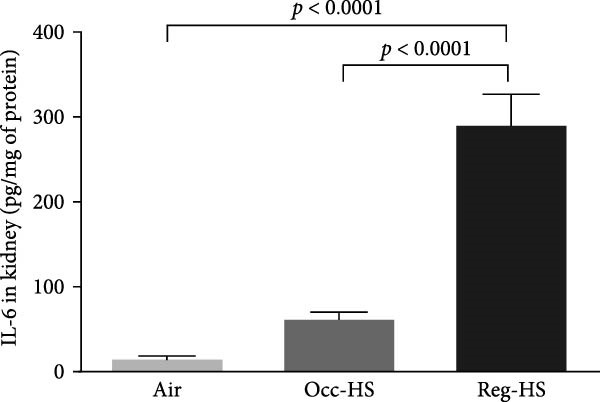
(B)

**Figure:**
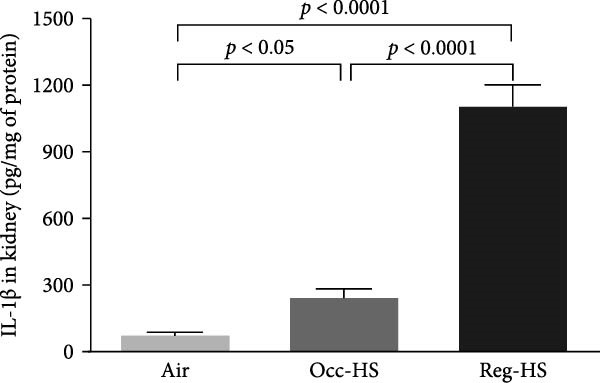
(C)

### 3.4. Concentrations of Renal Oxidative Stress Biomarkers

TBARS, nitric oxide, GSH, and SOD levels following exposure to either Occ‐HS or Reg‐HS are shown in Figure 4A–D. Our results revealed a significant elevation in TBARS (*p* < 0.05) and reduction in SOD (*p* < 0.001) following Occ‐HS inhalation. However, exposure to Reg‐HS led to an elevation in TBARS (*p*  < 0.0001) and reduction in the levels of nitric oxide, GSH, and SOD (*p* < 0.001–*p* < 0.001). Furthermore, there was a difference between Reg‐HS and Occ‐HS in the concentrations of TBARS, nitric oxide, and GSH (*p* < 0.01–*p* < 0.0001).

Figure 4Levels of thiobarbituric acid reactive substances (TBARS, A), nitric oxide (NO, B), reduced glutathione (GSH, C), and superoxide dismutase (SOD, D) in kidney tissue homogenates after 6 months of exposure to either air (control), occasional hookah smoke (Occ‐HS), or regular HS (Reg‐HS). Data are presented as mean ± SEM (*n* = 8).

**Figure:**
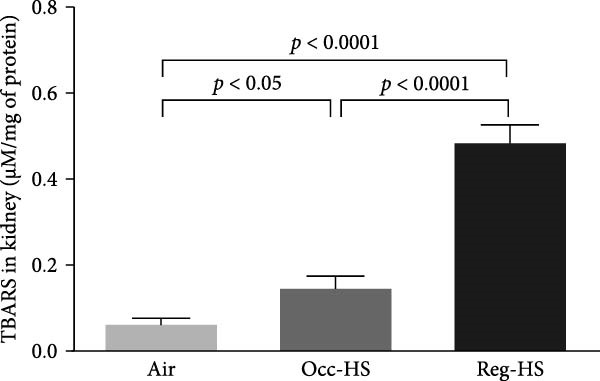
(A)

**Figure:**
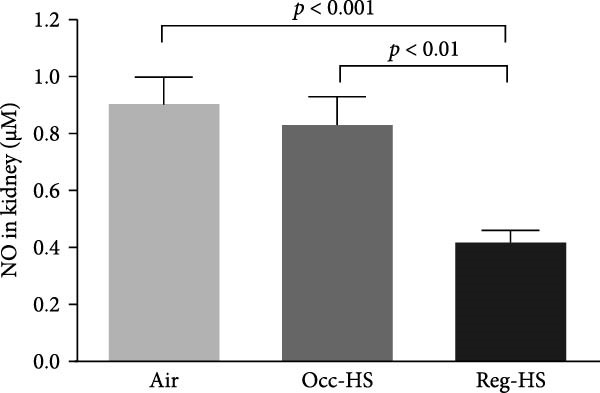
(B)

**Figure:**
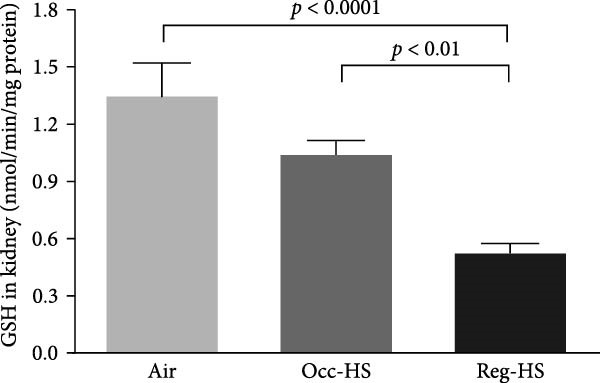
(C)

**Figure:**
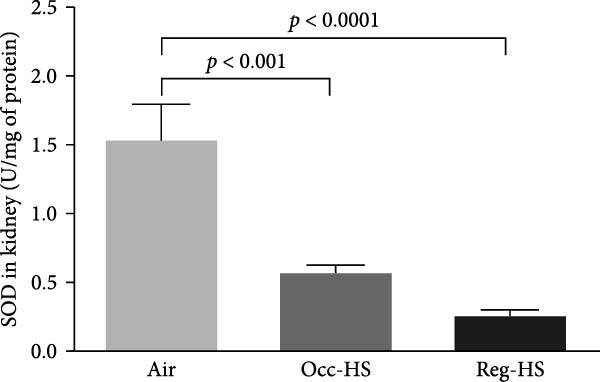
(D)

### 3.5. Renal Levels of 8‐OHdG

**Figure 5:**
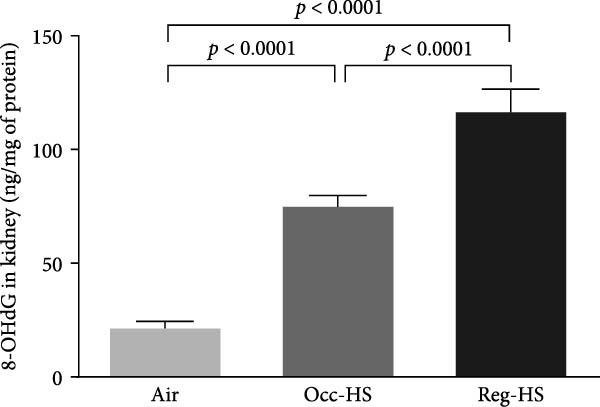
Concentration of 8‐Hydroxy‐2^′^‐deoxyguanosine (8‐OHdG) in kidney tissue homogenates after 6 months of exposure to either air (control), occasional hookah smoke (Occ‐HS), or regular HS (Reg‐HS). Data are presented as mean ± SEM (*n* = 8).

As illustrated in Figure 5, compared with air group, kidney tissue homogenates from mice exposed to either Occ‐HS or Reg‐HS for a duration of 6 months exhibited a marked increase in 8‐OHdG (*p* < 0.0001). Moreover, a statistically significant difference was observed between the Reg‐HS and Occ‐HS exposure groups (*p* < 0.0001).

### 3.6. Enzymatic Activities of Mitochondrial Electron Transport Chain Complexes I, II and III, and IV in Renal Tissue Homogenate

Exposure to either Reg‐HS or Occ‐HS resulted in a statistically significant increase in the activity of mitochondrial complex I, complexes II and III, as well as complex IV in renal tissue (*p* < 0.001–*p* < 0.0001). Furthermore, comparison between the Occ‐HS and Reg‐HS groups revealed significant differences (*p* < 0.01–*p* < 0.0001) in the enzymatic activities of complex I, complexes II and III, and complex IV (Figure 6).

Figure 6Mitochondrial complexes I (A), II and III (B), and IV (C) activities in kidney tissue homogenates after 6 months of exposure to either air (control), occasional hookah smoke (Occ‐HS), or regular HS (Reg‐HS). Data are presented as mean ± SEM (*n* = 8).

**Figure:**
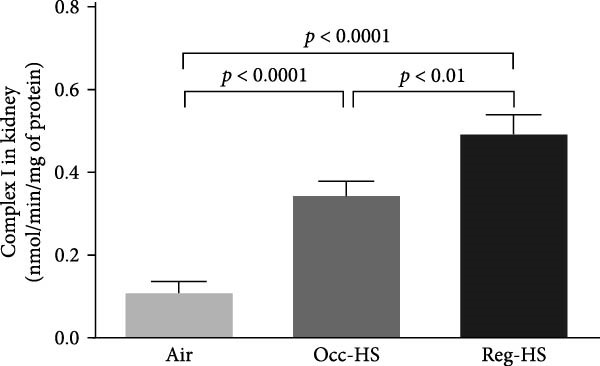
(A)

**Figure:**
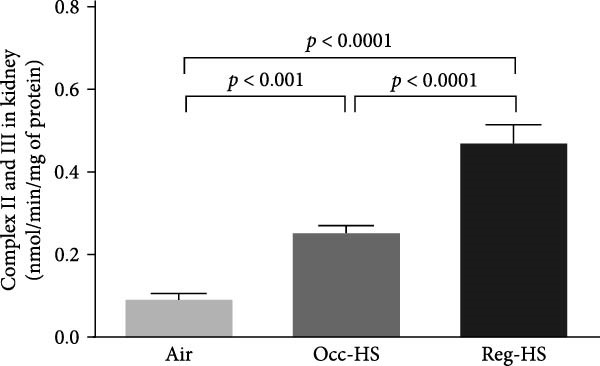
(B)

**Figure:**
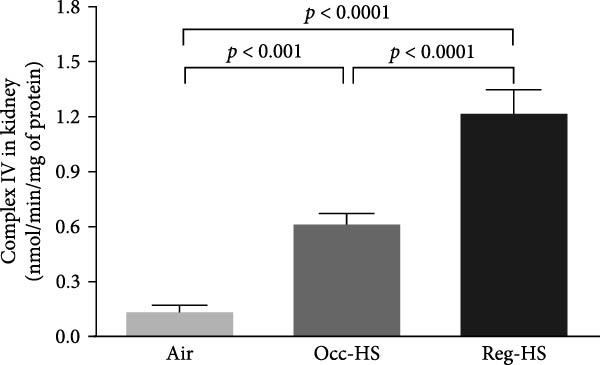
(C)

### 3.7. Expression of MAPK Kinases in the Kidney Tissue Homogenate

Figure 7 presents the western blot quantification of phosphorylated forms of JNK, ERK, and p38. No significant differences were observed in the expression of these proteins following Occ‐HS exposure relative to the control. In contrast, compared with the air group, Reg‐HS exposure caused a significant increase in the levels of p‐JNK, p‐ERK, and p‐p38 (*p* < 0.001–*p* < 0.0001). Furthermore, the Reg‐HS group exhibited significantly elevated expression of p‐JNK, p‐ERK, and p‐p38 compared to the Occ‐HS group (*p* < 0.001–*p* < 0.0001).

Figure 7Expression of phosphorylated (phospho) c‐Jun NH2‐terminal kinase (p‐JNK, A), extracellular signal‐regulated kinase (p‐ERK, B), and p‐p38 mitogen‐activated protein kinases (C) measured by western blot technique in kidney tissue homogenates after 6 months of exposure to either air (control), occasional hookah smoke (Occ‐HS), or regular HS (Reg‐HS). Data are presented as mean ± SEM (*n* = 8).

**Figure:**
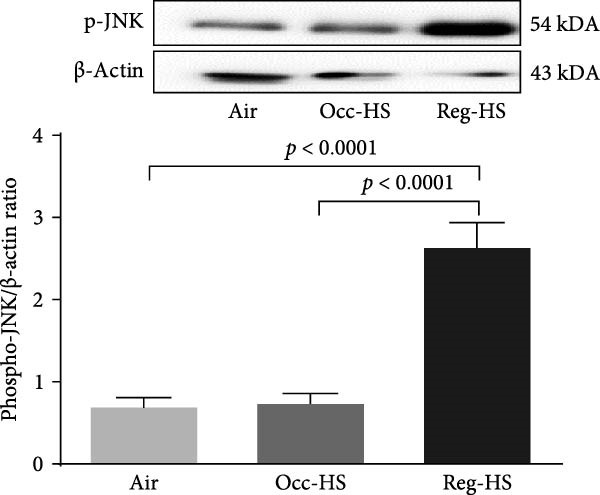
(A)

**Figure:**
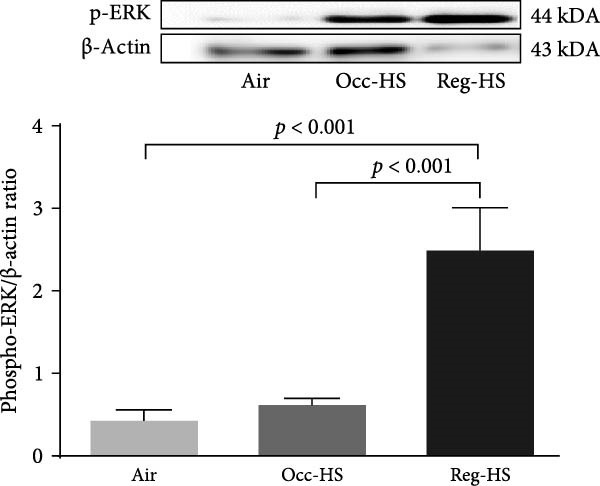
(B)

**Figure:**
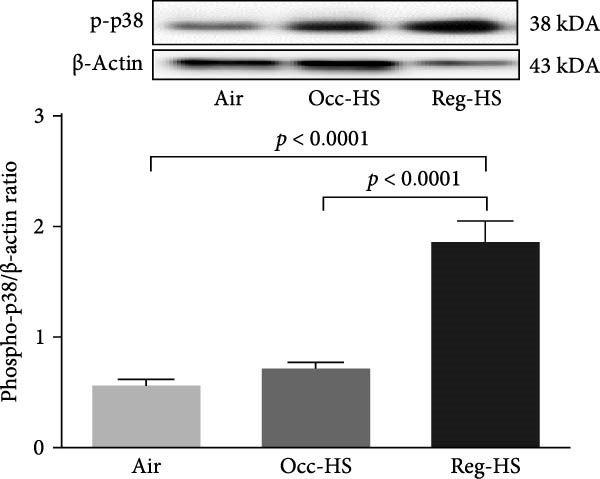
(C)

## 4. Discussion

By employing a validated nose‐only exposure model, we examined the effects of prolonged inhalation of either Occ‐HS or Reg‐HS on the kidney. Our findings provide experimental evidence that both regular and occasional inhalation of HS causes kidney injury. This is shown by an increase in classic kidney injury markers, alongside biochemical changes that manifest as oxidative stress, inflammation, and DNA damage. Additionally, it leads to mitochondrial dysfunction and alterations in the MAPKs signaling pathway.

We have found an increase in plasma creatinine and urea levels following exposure to either Occ‐HS or Reg‐HS. Our results align with previously reported data indicating that subchronic and chronic HS exposure resulted in an increase in creatinine, urea levels, and proteinuria as well as a decrease in clearance of creatine [20, 31, 32]. Notably, passive cigarette smoking has been shown to augment serum creatinine and decrease creatinine clearance rate in rats [16]. Additionally, a clinical study revealed elevated concentrations of creatinine and urea in the blood samples collected from cigarette smokers compared to nonsmokers [15].

Indoxyl sulfate is frequently measured in the bloodstream during kidney failure [33]. Indoxyl sulfate is linked to tubular injury, renal fibrosis, and is known to induce oxidative stress [34]. In our previous study, we showed that regular exposure to HS for 4 weeks did not elevate the concentration of indoxyl sulfate in the plasma [21]. However, in the current study a chronic exposure to Reg‐HS increased the concentration of indoxyl sulfate but not in Occ‐HS group.

The changes in biochemical parameters of blood analysis provide subsidies to understand the possible damage to kidney function. For that, this study aimed to elucidate the impact of either Occ‐HS or Reg‐HS inhalation on KIM‐1, a glycoprotein predominantly expressed in the epithelium of renal tubules of the kidney that plays a role in regulating immune cell activity as well as helps retaining albumin [35, 36]. NGAL, also described as lipocalin‐2, a small glycoprotein helps in the transportation of different molecules [37]. Increased levels of NGAL in plasma and urine are associated with kidney injury [38]. A significant upregulation of KIM‐1 and NGAL levels in renal tissue was observed following both Occ‐HS and Reg‐HS exposure. This indicates that even a short exposure, can lead to detrimental effects comparable to those observed with regular HS exposure. Recently, we have studied that regular acute and subchronic (4 weeks) HS exposure resulted in elevated levels of KIM‐1 [20]. In addition, urinary NGAL concentrations were significantly increased in mice following chronic whole‐body exposure to CS [17]. Clinically, urinary excretion of KIM‐1 and NGAL shown to be significantly higher among smokers than in nonsmoking individuals [18]. Moreover, chronic exposure to nicotine, showed an increase in KIM‐1 concentration [39, 40].

Serum cystatin C has become a well‐established biological marker for assessing kidney function and is considered to provide a more accurate measure of renal function compared to serum creatinine [41, 42]. This study revealed a nonsignificant rise in cystatin C concentration following Occ‐HS exposure, whereas Reg‐HS exposure resulted in a significant increase. These observations align with prior studies demonstrating elevated serum cystatin C levels in smokers relative to nonsmokers [43].

Cystatin C levels have been shown to have a strong correlation with inflammatory cytokine levels [44]. We have recorded a significant increase in the levels of IL‐1*β*, IL‐6, and TNF‐*α* following chronic exposure to Reg‐HS while Occ‐HS led to an elevation only in IL‐1*β* which can partially explain the proinflammatory properties of smoking. Our earlier findings demonstrated that 1 month of HS exposure led to a significant increase in the level of inflammatory cytokines [20, 21]. These findings are consistent with prior experimental studies that demonstrated CS exposure might contribute to a higher risk through production of inflammatory cytokines and oxidant radicals [45].

Inflammation and oxidative stress are widely recognized to be linked to various pathological conditions [46]. Proinflammatory cytokines, including TNF‐*α*, IL‐6, and IL‐1*β* can promote the generation of oxidants by activating several cellular pathways [47]. SOD is the major catalyst of superoxide dismutation, play a role in preventing the cellular formation of ROS and other free radicals [48, 49]. Oxidative stress plays a fundamental role in the development of renal injury, which is defined as depletion in the antioxidant capacity and an abundance of ROS which they are generated both as a natural consequence of metabolic pathways and under the influence of external stressors [50–52]. It is well established that overproduction of ROS contributes to pathological conditions and ultimately cell death [53].

Elevation in TBARS and reduction in SOD following Occ‐HS inhalation were observed in this study. However, exposure to Reg‐HS resulted in elevation in TBARS and reduction in the levels of nitric oxide, GSH, and SOD. These observations demonstrate that both Reg‐HS and Occ‐HS inhalation have significant oxidative stress effects by impairing multiple antioxidant pathways and increasing lipid peroxidation. These evidences reinforce the findings of our earlier research where we showed an increase in the levels of key oxidants, such as lipid peroxidation, ROS, and glutathione disulfide, and reduced levels of GSH, nitric oxide and catalase as well as increased SOD activity following subchronic Reg‐HS exposure in mice [20, 21]. The observed SOD increase in the previous study after 4 weeks could be associated with the overexpression of this enzyme in an attempt to overcome the oxidative effects of HS. However, in this study, following 6 months of exposure, A lowered level of SOD activity was observed, possibly due to the depletion of the kidney’s antioxidant defenses. Furthermore, our findings corroborate previous clinical and experimental studies reporting a decrease in antioxidant levels and exposure to CS or nicotine [17, 19, 39, 54–58].

It is well established that elevated ROS levels can affect biomolecules like nucleic acids, lipid membranes, and proteins which results in alteration in the cell function surpassing the antioxidant defenses [59–62]. In this study, we have observed that DNA integrity was compromised following exposure to either Occ‐HS or Reg‐HS through the increase of 8‐OHdG concentrations. These findings are coherent with prior results that showed the exposure to either HS or CS increase DNA oxidation in the kidney and lung [17, 20, 21, 32, 63].

We showed previously, that exposure to Reg‐HS and Occ‐HS has caused mitochondrial dysfunction in the kidney and heart [20, 23]. Consistently, we have observed in the present study, mitochondrial impairment in the kidney manifested by the increase of mitochondrial respiratory chain complexes in the kidney homogenates. In line with these findings, in vitro studies have reported mitochondrial alteration following exposure of human circulating lymphocytes, airway smooth muscle and epithelial cells to CS [64–66]. Findings from an in vivo study revealed that exposure to CS induced mitochondrial dysfunction [17].

To better understand the underlying mechanisms of toxicity induced by Occ‐HS and Reg‐HS inhalation, we further explored the potential downstream signaling events mediated by MAPKs activation would also be impacted by HS. In healthy tissue, the activation of the MAPKs pathways is primarily initiated by factors, such as metabolic stress, DNA damage, and cytokines which collectively influence cell viability [67]. Our findings revealed that exposure to Reg‐HS led to enhanced expression levels of p‐JNK, ap‐p38, and p‐ERK, in the kidneys, which is consistent with our previously published results [21].

This study has some limitations, the investigation lacks detailed mechanistic insights into how HS, particularly Occ‐HS vs. Reg‐HS exposure, contribute to the reported deleterious outcomes. Additionally, more research is required to identify the specific toxic substances implicated in the observed responses, with consideration of flavor‐specific toxic effects. Future research is necessary to address these gaps.

In conclusion, this study is the first to reveal that, despite notable differences in the amount of smoke inhaled, both regular and occasional HS exposure worsened kidney damage and triggered, cellular oxidative imbalance, inflammatory responses, and DNA integrity disruption as well as mitochondrial dysfunction in the kidneys of mice. Our findings present evidence that challenges the widely accepted belief that there is a safe level of HS, highlighting the need for increased awareness about the harmful effects related to the rising use of this tobacco consumption method. To effectively mitigate the risks posed by HS, we recommend that hookah smokers aim for complete cessation rather than simply reducing their consumption.

## Conflicts of Interest

The authors declare no conflicts of interest.

## Funding

The financial support for this research was provided through competitive grants awarded by the College of Medicine and Health Sciences (Grant 12M167) and the United Arab Emirates University and the Zayed Center for Health Sciences (Grant 12R166).

## Data Availability

Access to the underlying data can be granted by the corresponding author upon reasonable request.
